# Ensuring Adherence to 2023 National Institute of Excellence (Nice) Guidelines for CT Scans in Head Injury Cases in a Teaching Hospital in Basra, Iraq: A Clinical Audit

**DOI:** 10.7759/cureus.67282

**Published:** 2024-08-20

**Authors:** Hussein S Sadeq, Ahmed S Atwan, Sarmad R Alhilfi

**Affiliations:** 1 Department of Radiology, Basra Teaching Hospital, Basra, IRQ; 2 Department of Surgery, College of Medicine, University of Basra, Basra, IRQ

**Keywords:** neuroimaging, guideline adherence, traumatic brain injury, ct scan, clinical audit

## Abstract

Objective

The objective of this audit was to find out whether brain CT scans performed on patients with head trauma in Basra Teaching Hospital (BTH) adhere to the 2023 National Institute of Excellence (NICE) guidance for head injury (NG232) and whether we can improve this with selected interventions.

Methodology

We performed a clinical audit in two cycles; in the first cycle, we collected data retrospectively over a month in February 2024. The data was sourced from the imaging request forms and patient records at BTH. We then analyzed the data and implemented four key interventions to improve the outcome. After that, we performed our second audit cycle over an additional 30-day period during April 2024.

Results

Cycle One involved 59 patients, while Cycle Two involved 46. There was a significant decrease in scans requested outside of the NICE guidance, from 59.3% in Cycle One to 17.4% in Cycle Two (p<0.05). We also noticed a significant increase in the one-hour indication scans, from 32% in Cycle One to 65.2% in Cycle Two (p<0.05).

Conclusion

Our study findings reveal that by following some simple interventions, we significantly improved the adherence of our emergency department to the 2023 NICE guidelines for head CT following head trauma.

## Introduction

Traumatic brain injury (TBI) occurs when an external force, such as a bump, blow, or jolt to the head or body or an object penetrating the skull, alters brain function or causes other signs of brain pathology [1]. TBI represents a substantial global public health concern. In 2019, there were 27.16 million new cases of TBIs, leading to urgent medical visits and hospital admissions [2,3]. Additionally, during the same period, there were 48.99 million prevalent cases of TBI worldwide [2].

Low- and middle-income countries shoulder a substantial burden of TBI, with an estimated case count approximately three times higher than that observed in high-income countries [4]. Despite the scarcity of TBI literature in the Middle East and North Africa (MENA) region, there exists a disproportionately high prevalence of TBIs compared to the global burden. The incidence and severity of TBI exhibit remarkable variation across MENA countries, with mild TBI contributing significantly to the overall incidence proportion when compared to moderate and severe TBI cases [5].

CT scans serve as the gold standard for investigating head injuries due to their high sensitivity in detecting skull fractures and acute hemorrhages [6]. CT scans are more sensitive and specific than skull radiographs in detecting fractures and offer faster results at a lower cost compared to MRI scans [7,8]. Therefore, the National Institute for Health and Care Excellence (NICE) has provided guidelines to assist clinicians in managing TBI in the UK. In 2004, NICE replaced skull radiographs with CT as the primary diagnostic tool for head injuries. Subsequently, in 2014, updated NICE guidelines provided explicit criteria for CT scanning, establishing specific timeframes. This was amended in 2023 to improve the guidance further [9].

In Iraq, no specific guideline is designated for TBI imaging. Consequently, emergency medicine doctors often use international guidelines like NICE or the Canadian CT Head Rule (CCHR) to assess the need for imaging. This study aims to evaluate the number of CT scans ordered in Basra Teaching Hospital (BTH) in Basra, Iraq, in accordance with the NICE 2023 guidelines and whether certain interventions can improve our adherence to the guidance.

## Materials and methods

This clinical audit focused on assessing compliance with NICE guidelines regarding using CT head scans for head injuries in patients aged 16 years or older admitted to the emergency department (ED) of BTH. In alignment with Iraq's Ministry of Health guidelines, formal ethical approval is not required for clinical audits. However, the study received approval from the radiology department at BTH to ensure ethical considerations and adherence to institutional protocols.

The audited criteria are the 2023 NICE guidelines for doing a CT head scan for people 16 and over who have sustained a head injury (NG232), which are detailed in Table 1.

**Table 1:** Criteria for selecting people 16 and over for a CT head scan following a head injury according to NICE guidelines (NG232) GCS, Glasgow coma scale; NICE, National Institute of Excellence

CT head scan within one hour of the head injury if they have any of these risk factors:	CT head scans within eight hours of the head injury if they have a loss of consciousness or amnesia with any of these risk factors:
A GCS score of 12 or less on the initial assessment in the ED	Age 65 or over
A GCS score of less than 15 two hours after the injury on assessment in the ED	Any current bleeding or clotting disorders
Suspected open or depressed skull fracture	Dangerous mechanism of injury (a pedestrian or cyclist struck by a motor vehicle, an occupant ejected from a motor vehicle, or a fall from a height of more than 1 m or five stairs)
Any sign of basal skull fracture (hemotympanum, "panda" eyes, cerebrospinal fluid leakage from the ear or nose, Battle's sign)	More than 30 minutes retrograde amnesia of events immediately before the head injury
Post-traumatic seizure	
Focal neurological deficit	
More than one episode of vomiting	

The audit standard was the following: 100% of head CT requests must have documented head injury risk factor(s) justifying CT that comply with the 2023 NICE guidance.

In the first cycle of the audit, which we will refer to as Cycle One, a comprehensive retrospective data collection approach was employed. The data was collected over one month (February 2024). Non-head injury CT scans, such as those related to non-traumatic intracranial hemorrhage (ICH), strokes, or screenings for acute confusional states, were excluded from the analysis. Cycle One study examined a total sample size of 482 CT scans for patients admitted to the ED during the specified period, of which only 59 scans fit our inclusion criteria. The data was collected by radiologists and sourced from the imaging request forms and paper-based patient records from the ED at BTH. We recorded both the clinical indications for the scans and patient demographics. This sample size is more than the minimum number the Royal College of Radiologists advised for such studies [10].

**Figure 1:**
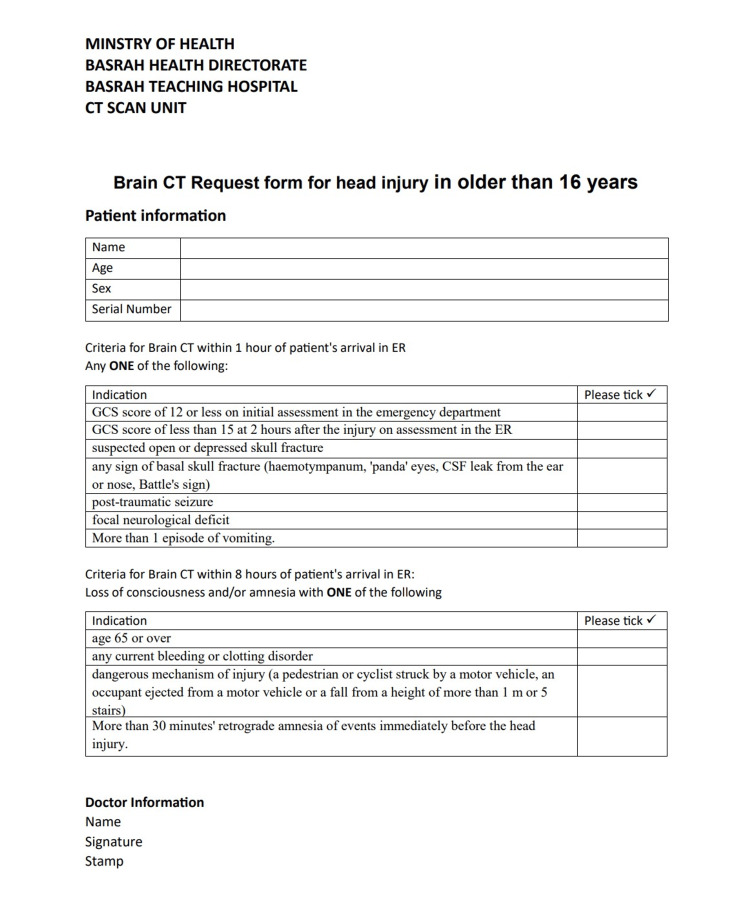
The newly introduced checklist-style form containing the NICE guidelines for head CT in trauma patients over 16 years of age NICE, National Institute of Excellence

After analyzing Cycle One results, the audit team, which included radiology and emergency medicine residents and a radiology consultant, discussed what changes could be implemented to improve adherence to the NICE guidance. The team agreed upon four essential interventions derived from similar studies in the literature and input from the radiology department. First, the initial study's findings would be shared with the teams operating in the ED, who are responsible for requesting CT scans, through a team meeting. Second, our hospital will adopt the 2023 NICE Guidelines for CT Scans in Traumatic Head Injury as the exclusive reference for managing TBI cases. Third, the request process will be streamlined by introducing a new checklist-style form for requesting CT head scans, aligning it with the updated NICE guidance as Figure 1 (in the Appendix). Finally, to reinforce adherence, emergency medicine residents and specialists working in the ED will be periodically educated about the guidelines through a combination of educational sessions and regular updates during departmental meetings.

After introducing changes, a second cycle of the audit, referred to as Cycle Two henceforth, was conducted. Data was collected over an additional 30 days (April 2024) to evaluate whether there was a significant improvement after applying the suggested strategies. We looked at 475 total scans, and only 46 were included in the study.

We analyzed the data using the IBM SPSS Statistics for Windows, Version 29.0.2.0, employing descriptive statistical methods such as percentages, means, standard deviations, and confidence intervals. Additionally, the Chi-square test was used to determine the significance of the associations. Statistical significance was set at a p-value of less than 0.05.

## Results

During Cycle One of the study period, 59 head CT scans were ordered for 16-year-old and over patients presenting to the ED with head injury. The study population exhibited a mean age of 33.7±16.65 years, with the youngest participant being 16 and the oldest 81. Notably, male patients constituted 74.5% of the study.

During Cycle Two, 46 head scans that fit our inclusion criteria were ordered in the 30 days. The second cycle mean age was 34±18.29 years, and the age range was 16-85, again showing a predominance of the male population with a percentage of 78.3% (Table 2).

**Table 2:** Patient demographics The data is presented as n(%).

Cycle One	Total	Male patients	Female patients
Number	59 (100%)	44 (74.5%)	15 (25.5%)
Average age (years) (mean±SD)	33.7±16.65	32.84±17.53	36.27±14.02
Cycle Two	Total	Male patients	Female patients
Number	46 (100%)	36 (78.3%)	10 (21.7%)
Average age (years) (mean±SD)	34±18.29	31.11±15.96	44.5±22.96

In Cycle One, 24 of the total scans adhered to the NICE criteria for the one- and eight-hour window. However, 35 scans were performed without a discernible clinical indication. This suggests that 59.3% of the requested scans were for indications that deviated from the NICE guidelines. Cycle Two, however, showed that 38 scans out of 46 were in line with the NICE guidelines. This indicates that only 17.4% of scans did not adhere to the audited guidance. This is a statistically significant decrease of 41.6% (95% confidence interval: 25.2%-58.5%) that occurred in Cycle Two following interventions (p<0.05).

Regarding the one-hour criteria, in Cycle One, 19 cases were documented to have one-hour indications, composing 32% of the total scans requested. In Cycle Two, 30 scans fulfilled the one-hour criteria, accounting for 65.2% of the requested scans. This shows an increase of 33.2% (95% confidence interval: 15%-51.4%) after the application of changes, which is significant (p<0.05).

In the context of the eight-hour criteria, Cycle One recorded five cases (8.4%), whereas Cycle Two recorded eight cases (17.4%). This indicates a 9% increase in Cycle Two, which is not statistically significant (p>0.05) (Table 3).

**Table 3:** Comparison between the performance of the two audit cycles Statistical analysis was performed using the Chi-square test, with significance set at p<0.05.

Cycle	Scans adherent to the guidelines	Scans not adherent to the guidelines	P-value	Scans with one-hour indications	Scans without one-hour indications	P-value	Scans with eight-hour indications	Scans without eight-hour indications	P-value
1	24	35	0.00001	19	40	0.00007	5	54	0.1687
2	38	8		30	18		8	38	

The documented one-hour and eight-hour indications for head CT after trauma in both cycles are detailed in Table 4 and Table 5, respectively. It is important to note that while these documented criteria provide insight, they do not necessarily account for instances where the criteria were assessed and communicated within the ED team but were not explicitly documented in our records.

**Table 4:** The documented one-hour indications for head CT after trauma in both cycles Indications documented are presented as n(%). Some cases have multiple indications documented, resulting in a higher number of indications than scans.

One-hour indications recorded	Cycle One (indications documented: 19)	Cycle Two (indications documented: 57)
GCS score of 12 or less on initial assessment in the emergency department.	5 (26.3%)	9 (15.8%)
GCS score of less than 15 two hours after the injury on assessment in the emergency department.	0 (0%)	6 (10.5%)
Suspected open or depressed skull fracture.	1 (5.3%)	10 (17.5%)
Any sign of basal skull fracture.	7 (36.8%)	7 (12.3%)
Post-traumatic seizure.	0 (0%)	5 (8.8%)
Focal neurological deficit.	1 (5.3%)	4 (7%)
More than one episode of vomiting.	5 (26.3%)	16 (28.1%)

**Table 5:** The documented eight-hour indications for head CT after trauma in both cycles Indications documented are presented as n(%). Some cases have multiple indications documented, resulting in a higher number of indications than scans.

Eight-hour indications documented loss of consciousness or amnesia with one of:	Cycle One (indications documented: 5)	Cycle Two (indications documented: 9)
			Age 65 or over	0 (0%)	3 (33.3%)
Any current bleeding or clotting disorder	0 (0%)	0 (0%)
Dangerous mechanism of injury	5 (100%)	6 (66.7%)
More than 30 minutes retrograde amnesia of events immediately before the head injury	0 (0%)	0 (0%)

Our observations regarding indications that do not align with the NICE guidance are detailed in Table 6.

**Table 6:** Documented indications that do not align with the NICE guidance Indications documented are presented as n(%). Some cases have multiple indications documented, resulting in a higher number of indications than scans. NICE, National Institute of Excellence

Indication	Cycle One (indications documented: 35)	Cycle Two (indications documented: 9)
Road traffic accident +- headache	13 (37.1%)	2 (22.2%)
Fall from a height	7 (20%)	0 (0%)
Nausea and/or vomiting once or not specified frequency	4 (11.4%)	1 (11.1%)
Dizziness	1 (2.9%)	0 (0%)
Transient loss of consciousness	1 (2.9%)	2 (22.2%)
Head trauma by a heavy object	1 (2.9%)	0 (0%)
No specific indication was mentioned apart from head trauma	8 (22.8%)	4 (44.5%)

## Discussion

While Brain CT scans play a crucial role in assessing TBI, it is equally essential to exercise caution and prevent excessive utilization of this imaging modality. CT imaging is a valuable diagnostic tool, yet it carries inherent risks. An adult undergoing a single-head CT scan receives a dose of radiation of two mSv, which corresponds to an incremental lifetime risk of cancer estimated at one in 10,000 for each scan performed [11].

In Cycle One of the audit, it was noticed that a significant proportion of patients undergoing CT scans did not align with the NICE guidance. Our proportion of 59.3% is higher than what was found in a study conducted at The John Radcliffe Hospital, Oxford, UK, which demonstrated non-adherence to guidelines at 15.7% [12]. This phenomenon may be attributed to several factors, including the threat of medical liability, challenges encountered when applying guidelines and documenting criteria within medical records, difficulty in changing the current practice due to fear of judgment from colleagues, and doubts regarding the credibility of the evidence or research supporting the recommendations [13-15]. Our findings, however, are consistent with the findings of a study conducted in Al-Hussin Educational Hospital, Iraq, that showed 41% of the scans performed in the ED were unjustified according to NICE guidance, indicating the necessity of similar studies and quality improvement projects focusing on this field in our region [16].

An observation of note is that the predominant indication documented for CT scans not adhering to the NICE guidelines was dangerous mechanisms of injury, including road traffic accidents or falls, without any additional rationale, such as loss of consciousness or amnesia. This trend suggests a prevalent misunderstanding that accidents alone are sufficient grounds for a head CT scan, a notion that is inconsistent with our reviewed guidelines. Routine ordering of head CT scans for adults who have traumatic accidents is inefficient, particularly in older age groups, as most of these patients do not exhibit intracranial bleeding. Additionally, the cost of such scans can burden hospital resources. Overutilization of CT scans in older people also leads to prolonged ED length of stay, which, in turn, has been associated with an increased incidence of delirium. Furthermore, this practice diverts valuable time and effort from other emergency patients [17].

Our study findings reveal that the strategies implemented in Cycle Two have been successful. We significantly decreased the number of patients who underwent head CT due to indications that fell outside of the NICE guidance. Concurrently, we increased the number of CT scans done for the one-hour indication, which is essential due to the time-sensitive nature of these cases. A similar study at the Royal Free Hospital in London implemented similar interventions and showed an increase in adherence to NICE guidelines after the introduction of changes [6]. This success suggests that these measures are a reliable way to improve the effectiveness of similar clinical audits, instilling optimism for the potential for improvement.

Although we did not achieve our standard goal of 100% adherence to the NICE guidance, we have succeeded in improving our practice in a relatively short period. Therefore, maintaining these changes and incorporating more strategies are necessary to achieve the desired quality target.

Limitations

This clinical audit acknowledges the limitations imposed by its relatively small sample size. It is also limited by the fact that we do not know whether this improvement is a short-lived change due to knowledge of the audit efforts or a sustainable change that will persist after the completion of our study. Therefore, it is crucial that we continue to review and evaluate our practices to ensure sustained improvement.

Additionally, the study recognizes that some patient information, which could have justified the CT scan requests, may have been shared verbally among the ED team members but was not documented, leading to some scans being incorrectly categorized as "Indications that do not align with the NICE guidance." Furthermore, although we introduced the new checklist-style request form, we still had requests that followed the traditional form and requests that did not have documented indications, which means the transition to the new form could have been more seamless. This highlights the need for additional training for the ED staff, emphasizing the importance of continuous learning and improvement.

## Conclusions

This clinical audit showed that using simple means and interventions significantly improved our compliance with the 2023 NICE guidelines for head CT in trauma patients visiting BTH. This has successfully reduced the number of patients exposed to unnecessary scans. However, to improve our practice further and achieve the standard of 100% adherence, work is still needed to incorporate more changes and maintain the current ones. Further projects are also required to continue the process of quality improvement.
